# Improved Reconstruction Quality of Bioluminescent Images by Combining SP_**3**_ Equations and Bregman Iteration Method

**DOI:** 10.1155/2013/767296

**Published:** 2013-01-22

**Authors:** Qiang Wu, Jinchao Feng, Kebin Jia, Xiangyu Wang

**Affiliations:** College of Electronic Information and Control Engineering, Beijing University of Technology, Beijing 100124, China

## Abstract

Bioluminescence tomography (BLT) has a great potential to provide a powerful tool for tumor detection, monitoring tumor therapy progress, and drug development; developing new reconstruction algorithms will advance the technique to practical applications. In the paper, we propose a BLT reconstruction algorithm by combining SP_3_ equations and Bregman iteration method to improve the quality of reconstructed sources. The numerical results for homogeneous and heterogeneous phantoms are very encouraging and give significant improvement over the algorithms without the use of SP_3_ equations and Bregman iteration method.

## 1. Introduction

As an emerging molecular imaging technique, bioluminescence imaging (BLI) is potentially well suited for early detection, clinical drug development and monitoring, and regeneration research [1–5]. Therefore, this imaging modality has received increasingly intense research interest worldwide over the recent years.

To date, planar BLI is commonly used because of its ease of implementation and operational simplicity, but it also suffers from significant limitations, including the low resolution, the lack of quantification, and the incapacity of accurately providing depth information [6]. In contrast, bioluminescence tomography (BLT) could overcome these limitations by using accurate reconstruction algorithms coupled with theoretical models of photon propagation in biological tissues, providing higher resolution, quantification accuracy, and depth information [7]. In comparing BLT to planar BLI, planar BLI is a qualitative analysis and BLT is a quantitative analysis [8]. Therefore, scientists are now paying more attention to the advancement of BLT research.

The objective of BLT is to recover the unknown bioluminescent source distribution *s* ∈ ℝ^*n*^ based on the noisy surface measurements Φ^meas⁡^ ∈ ℝ^*m*^ [6, 7]. Indeed, the problem is also called the inverse problem. However, a major difficulty in recovering the bioluminescent source distribution is imposed by multiple scattering which occurs when light propagates through biological tissues. This makes the inverse problem severely ill-posed [7]. Furthermore, the number of recovered unknown source distributions is usually far more than the number of detected boundary measurements, that is, *m* < *n* (in many cases, *m* ≪ *n*). Hence, BLT is also a typically underdetermined problem. To obtain a meaningful solution, regularization techniques are usually adopted, which consist of solving the following constrained optimization problem [9]:

(1) $$\begin{array}{c} \underset{s\geq 0}{\min}{||As-\Phi^{\mathrm{meas}}||}_{2}^{2}+\lambda \cdot J\left(s\right), \end{array}$$

where *J*(·) is a properly chosen regularization term, *λ* > 0 represents regularization parameter, and *A* ∈ ℝ^*m*×*n*^ is a linear operator, typically formed by discretizing diffusion equation with finite element methods [10].

When *J*(*s*) = ||*s*||_2_^2^, the above regularized problem becomes the popular Tikhonov regularization, which inherently provides smoothed solutions and therefore offers compromised accuracy in localizing bioluminescent sources [11]. Recently, *l*_1_-regularized problems, that is, *J*(*s*) = ||*s*||_1_, have received an increasing amount of attention in optical imaging, which allow high-quality images to be reconstructed from a small amount of boundary measurements [11–14]. However, *l*_1_-regularized problems can sparsify the bioluminescent source distribution, which affects the quality of reconstructed images [13, 15].

Furthermore, in order to obtain the matrix *A* in (1), the diffusion approximation (DA) to radiative transfer equation (RTE) is widely used as the forward model for BLT reconstructions. Although the DA is one of the most important approximation methods in BLT [6–11], it suffers from some limitations [12–14]. Firstly, the scattering is dominated over absorption and secondly, the DA fails in modeling light propagation in the vicinity of those highly vascularized tissue parts [12–14]. Therefore, the DA will introduce significant error in some BLT cases [14]. In contrast, the RTE is widely accepted as an accurate model for light propagation in biological tissues. However, the use of the RTE as the forward model for BLT is often not feasible due to the facts that analytical solutions cannot exist for biological tissues with spatially nonuniform scattering and absorption properties and the computation of numerical approximations for the solution is extremely time consuming [16, 17]. A generalized delta-Eddington phase function was recently presented to simplify the RTE, and the more accurate solution was obtained relative to the DA [18, 19]. However, the parameter *f* used in the model is difficult to compute [18, 19]. In addition, the system matrix for the model is also difficult to construct for complex heterogeneous geometries. These factors seriously limit the utilization of the model in BLT. The use of simplified spherical harmonics (SP_N_) equations to approximate the RTE has been demonstrated to significantly improve the diffusion solution in domains with high absorption and small geometries [5, 12–14, 16, 20]. Meanwhile, the SP_N_ methods are computationally less expensive than the RTE ones. 

Large efforts in combining multiple types of *a priori* information to develop BLT reconstruction algorithms to improve the quality of reconstructed images, particularly the permissible source region and multispectral information, have formed the grounds of BLT reconstructions [9–11, 20–26]. Despite the recent advances in BLT reconstruction algorithms and light propagation models, it is necessary to develop and refine reconstruction methods to improve image quality.

Bregman iteration method has been studied recently and is widely used in compressed sensing [27, 28]. The idea is to add the residual, that is, the error produced at the current iteration, back to the data for the next iteration to be corrected [27]. The method is particularly attractive for sparse reconstruction, but so far it has not been fully investigated and analyzed in BLT, and this is the goal of this paper.

In this paper, we propose a BLT algorithm to improve the quality of reconstructed images. In the algorithm, SP_3_ equations are adapted to model light propagation, and Bregman iteration method is used to solve the inverse problem for BLT. Numerical results demonstrate that the quality of reconstructed images is improved greatly. The rest of the paper is organized as follows. In the following section, we described SP_3_ equations as light propagation model and Bregman iteration method. Last, numerical experiments were performed to evaluate the proposed algorithm, and corresponding conclusions were made. 

## 2. Methods

### 2.1. SP_3_ Equations

The propagation of light in biological tissues can be well modeled by SP_3_ equations. SP_3_ equations are two coupled diffusion equations for the moments *ϕ*_1_ and *ϕ*_2_ [16, 17]:

(2) −∇·(13μa1(r)∇ϕ1(r))+μa(r)ϕ1(r)−2μa(r)3ϕ2(r)=S(r),−2μa(r)3ϕ1(r)−∇·(17μa3(r)∇ϕ2(r))   +(49μa(r)+59μa2(r))ϕ2(r)=−23S(r),

where *μ*_*an*_ = *μ*_*a*_ + (1 − *g* ^*m*^) · *μ*_*s*_ (*m* = 1,2, 3), and *μ*_*a*_ and *μ*_*s*_ are the absorption and scattering parameters, respectively. *g* is the anisotropy parameter.

The boundary conditions are given by

(3) (12+A1)ϕ1(r)+1+B13μa1(r)(n·∇ϕ1(r))  =(18+C1)ϕ2(r)+D1μa3(r)(n·∇ϕ2(r)),(724+A2)ϕ2(r)+1+B27μa3(r)(n·∇ϕ2(r))  =(18+C2)ϕ1(r)+D2μa1(r)(n·∇ϕ1(r)).

The coefficients *A*_1_,…, *D*_1_,…, *A*_2_,…, *D*_2_ can be found in [16]. Furthermore, the partial current can be obtained from solutions *ϕ*_1_ and *ϕ*_2_:

(4) J+(r)=(14+J0)ϕ1(r)−0.5+J13μa1(r)(n·∇ϕ1(r))+(−116−23J0+13J2)ϕ2(r)−J37μa3(r)(n·∇ϕ2(r)).

The coefficients *J*_0_, *J*_1_,…, *J*_3_ can also be found in [16]. Solving the above equations by finite element methods, a linear operator *A* can be established [29].

### 2.2. Bregman Iteration Method

Bregman iteration method is based on the definition of Bregman distance. The Bregman distance associated with a convex function *E* at the point *υ* is given as [27]

(5) $$\begin{array}{c} D_{E}^{p}\left(u,v\right)=E\left(u\right)-E\left(v\right)-\langle p,u-v\rangle , \end{array}$$

where *p* ∈ ∂*E* is in the subgradient of *E* at *v*. Clearly, this is not a distance in the usual sense because it is not in general symmetric. However, it does measure closeness in the sense that *D*_*E*_^*p*^(*u*, *v*) ≥ 0 and *D*_*E*_^*p*^(*u*, *v*) ≥ *D*_*E*_^*p*^(*w*, *v*) for *w* on the line segment between *u* and *v* [27].

Based on Bregman iteration method, (1) can be reformulated as

(6) sk+1=arg min⁡s≥0⁡DJp(s,sk)+1λ||As−Φmeas⁡||22=arg min⁡s≥0⁡{J(s)−〈pk,s−sk〉+1λ||As−Φmeas⁡||22},pk+1=pk−1λAT(Ask+1−Φmeas⁡),

where *p*_*k*+1_ ∈ ∂*E*(*s*_*k*+1_) and *A* ^*T*^ is the adjoint operator of *A*. Since the operator *A* is linear in BLT reconstructions, the above complicated iteration can be transformed to the following two-stage iteration procedure with *v*_0_ = 0 [27]:

(7) $$\begin{array}{c} s_{k+1}=\arg\;\underset{s\geq 0}{\min}\left\{{||As-\left(\Phi^{\mathrm{meas}}+v_{n}\right)||}_{2}^{2}+\lambda \cdot J\left(s\right)\right\}, \end{array}$$

(8) $$\begin{array}{c} v_{k+1}=v_{k}+\Phi^{\mathrm{meas}}-As_{k+1}. \end{array}$$

This is done by iteratively solving the optimization problem (7) and then modifying the measured value of Φ^meas⁡^ used in the next iteration. And (8) is usually referred as “adding back the noise” [30]. In the paper, *J*(·) is fixed as the *l*_1_ regularizer. The implementation of (7) was performed by a gradient projected (GP) algorithm [31]. The proposed algorithm was depicted in Algorithm 1.

## 3. Results

To fully evaluate the performance of the proposed algorithm, homogeneous and heterogeneous experiments were performed. In the experiments, the parameters *ε* and *k*_max⁡_ were set to 1 × 10^−3^ and 10, respectively. The parameters in GP algorithm set default values, except the maximum iteration number is fixed at 50000 to ensure the convergence of the algorithm unless otherwise is specified.

### 3.1. Homogeneous Phantom Experiments

**Figure 1:**
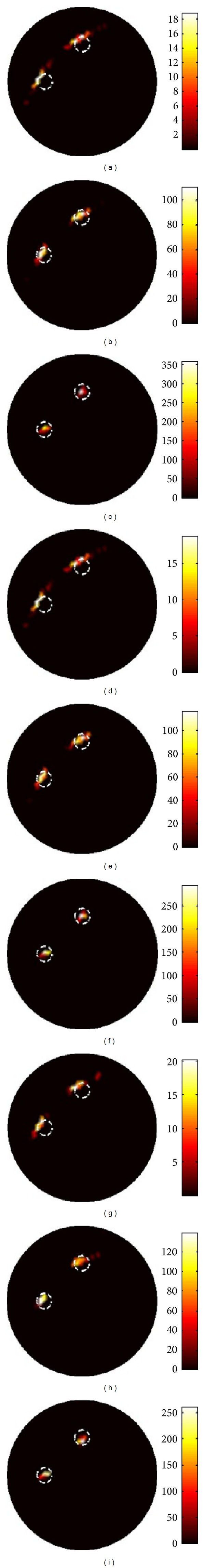
Reconstructed images with different methods with different levels of noisy data. The first and second columns are reconstructed results with the DA and SP_3_ equations as the forward models, respectively. The last column is the images by combing SP_3_ equations and Bregman iteration method. The first row is the results with noise-free data and the middle and last rows are the results with 10% and 30% noisy data. The white circles represent the actual sources.

In this section, 2D numerical simulations were used to investigate the performance of the proposed algorithm since less computational time was required for 2D data. Here, two individual cases were considered. In the first case, numerical simulations were performed on a homogenous circle with 10 mm radius. Within this circle, two sources (source 1 and source 2) were placed in (−5, 0) mm and (0, 5) mm, respectively and each source had a radius of 1.0 mm. The corresponding optical parameters were listed in Table 1. The boundary data were generated for two wavelengths (600 and 620 nm) with finite element methods, and different levels of Gaussian noise (0%, 10%, and 30%) were added to the datasets. BLT reconstructions were performed without and with Bregman iteration method. Corresponding results were shown in Figure 1. In this case, the ratios of *μ*_*s*_′/*μ*_*a*_ are larger than 10; therefore, the circular phantom has high-scattering characteristics. Hence, the DA is suitable for the simulation. For comparison, we carried out BLT reconstructions with the DA as the forward model; reconstructed images were also illustrated in Figure 1. From Figure 1, we can see that the results with SP_3_ equations are better than those obtained with the DA and Bregman iteration method can improve the quality of reconstructed images. The best results are obtained by combing SP_3_ equations and Bregman iteration method. In addition, quantitative results were summarized in Table 2. Data in Table 2 show that reconstructed position errors can be significantly reduced when SP_3_ equations are used together with Bregman iteration method.

**Figure 2:**
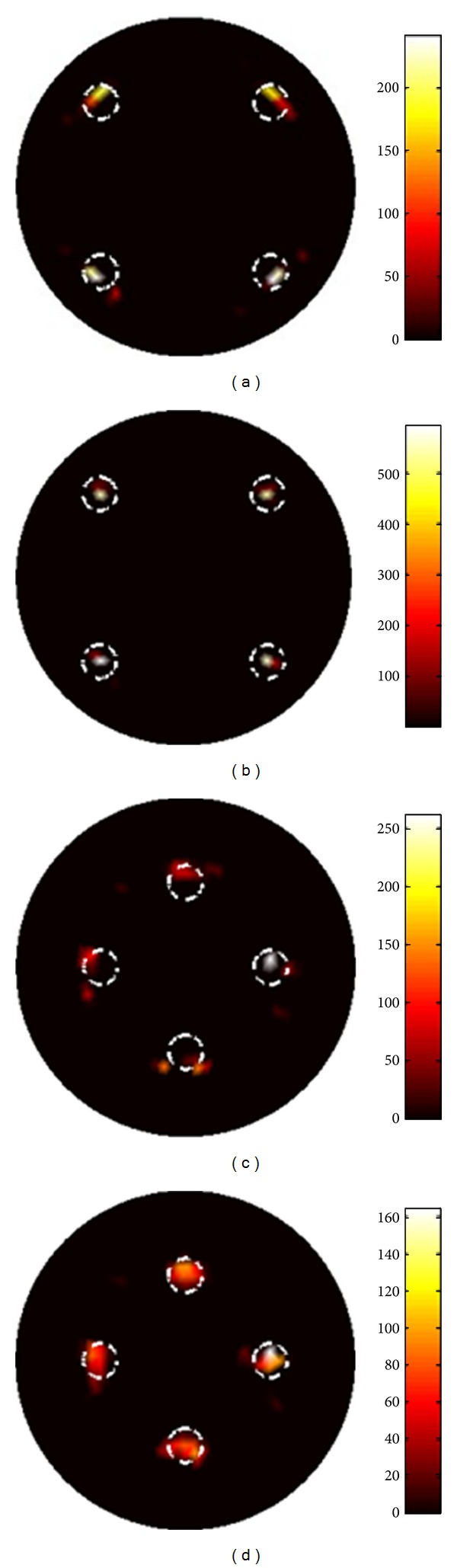
Reconstructed images in the case of four sources. The corresponding images are shown for sources near the surfaces (top row) and near the center (bottom row). (a) and (c) are results obtained only with SP_3_ equations. (b) and (d) are the results by combing SP_3_ equations and Bregman iteration method.

Furthermore, we tested the proposed algorithm by using experiments with multiple bioluminescent sources. The optical properties of a real mouse muscle for different wavelengths (580 and 620 nm) were assigned as listed in Table 3 [29]. Four identical sources with 1 mm radii were placed different positions. First, the sources were placed near the surfaces, and the distance to the center of the circle was 7.07 mm. The boundary measurements were also produced by finite element methods, and 20% Gaussian noise was added into the simulated data. Note that in the test, *μ*_*s*_′/*μ*_*a*_ for two wavelengths are less than 10; therefore, the condition *μ*_*s*_′ ≫ *μ*_*a*_ does not hold and the DA is less valid. Hence, BLT reconstructions with the DA were not implemented. The results with SP_3_ equations are shown in Figures 2(a) and 2(b). Next, the sources were placed at 5 mm positions off the center. Then BLT reconstructions were performed, as shown in Figures 2(c) and 2(d). Furthermore, quantitative results were shown in Table 4. It is worthy of mentioning that BLT reconstructions without and with Bregman iteration method use the same regularization parameter (i.e., 3 × 10^−6^), but the reconstructed results are different. From Figure 2 and Table 4, it is easily concluded that better images can be obtained by combining SP_3_ equations and Bregman iteration method.

### 3.2. Heterogeneous Phantom

**Figure 3:**
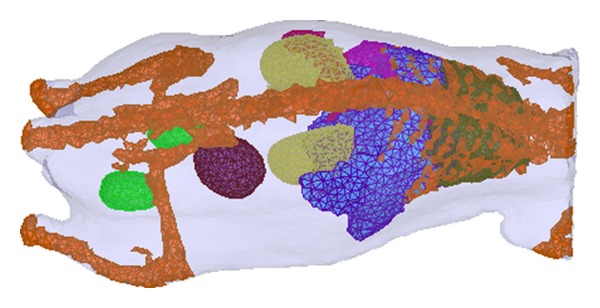
The heterogeneous mouse phantom.

**Figure 4:**
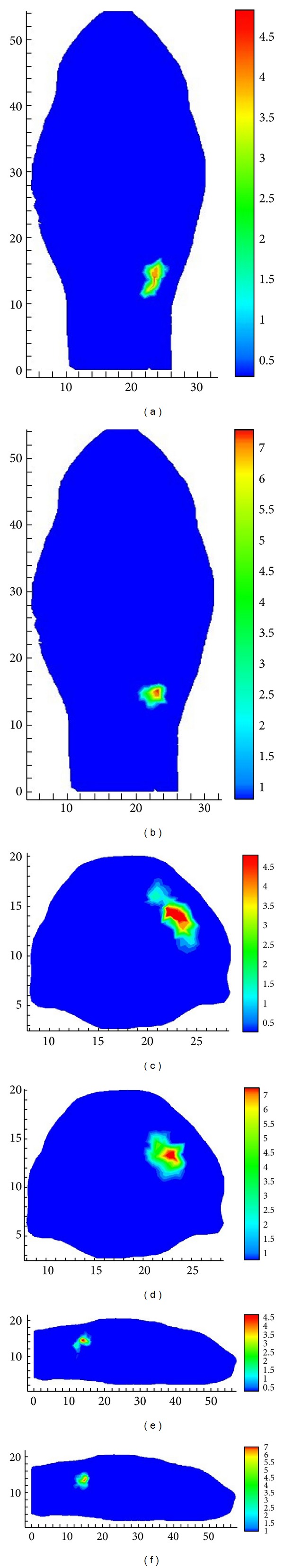
Cross sections of the reconstructed images through the actual center of the real source for heterogeneous mouse experiment. (a) and (b) are coronal sections; (c) and (d) transverse sections; (e) and (f) sagittal sections. The first and second columns show reconstructions without and with Bregman iteration method, respectively.

In the subsection, a micro-MRI-based heterogeneous mouse model (MOBY) was used to validate the proposed algorithm [32]. About 2/3 of the entire phantom was used for mesh generation, and a volumetric mesh with 17661 nodes and 93312 tetrahedron elements was obtained by iso2mesh [33], as shown in Figure 3. The optical properties of different tissues were assigned according to Table 5, reproduced from Alexandrakis et al. [21]. The forward simulation data was produced by finite element methods, and 10% Gaussian noise was added. Then BLT reconstructions were performed without and with Bregman iteration method. The regularization parameters used in the two methods were the same, and the value was 0.1. The maximum iteration number in the GP algorithm was set to 5000, and other parameters remained unchanged. The reconstructed results without and with Bregman iteration method were shown in Figure 4. From the images, we can see that the quality of reconstructed images can be improved with the use of Bregman iteration method. Furthermore, the reconstructed central positions for the two algorithms are (22.77, 14.95, 13.33 mm) and (22.24, 13.95, 14.49 mm), respectively. The real source position is (22.07, 14.43, 13.06 mm). The absolute distances between reconstructed sources and the real source are 0.91 mm and 1.52 mm, respectively. The quantitative results also demonstrate that Bregman iteration method can improve the quality of reconstructed images. 

## 4. Conclusion

We have presented a BLT reconstruction algorithm by combing SP_3_ equations and Bregman iteration method as a competitive method for reconstructing bioluminescent sources and validated the proposed algorithm using homogeneous and heterogeneous experiments. It has been demonstrated that the proposed algorithm can enhance the recovery of bioluminescent sources in terms of the quality of reconstructed images and localization error. 

The use of SP_3_ equations is a helpful technique to improve BLT reconstructions. Our experiments have illustrated that the appearance of artifacts can be reduced when SP_3 _equations are used as the forward model. However, the computation of the system matrix *A* by solving SP_3_ equations is very expensive, especially when the imaged objects are very complex, irregular, and heterogeneous. Fortunately, with the fast development of graphics processing unit (GPU), the computation of *A* can be significantly accelerated.

One merit of the proposed algorithm is that the improved results are obtained by making use of the available boundary measurements and thus do not require increased number of boundary measurements and do not bring more hardware requirements. Meanwhile, the proposed algorithm is relatively easy to implement. Therefore, the algorithm is suitable for *in vivo* applications. As a sacrifice, the computational burden for the proposed algorithm is greatly increased, especially for the heterogeneous mouse experiment, since solving (1) brings extra cost through Bregman iteration method, and each iteration of which is equivalent of solving a standard “L1” problem. To increase computational efficiency for mouse experiments, developing fast large-scale optimization algorithms is essential. 

In conclusion, we have developed a BLT reconstruction algorithm by combing SP_3_ equations and Bregman iteration method and indicated its feasibility and merits. In the near future, we expect to accelerate the proposed algorithm based on GPU and extend it to *in vivo* mouse experiments.

## Figures and Tables

**Algorithm 1:**
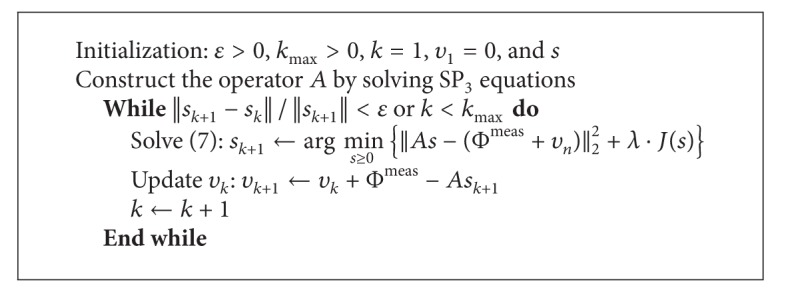
BLT reconstruction with SP_3_ equations and Bregman iteration method.

**Table 1:** Optical properties for different bands [22].

Wavelength	*μ*_*a*_ (mm^−1^)	*μ*_*s*_^'^ (mm^−1^)	*μ*_*s*_^'^/*μ*_*a*_	*g*
600 nm	0.0281	1.6667	59.3	0.9
620 nm	0.0109	1.6129	147.9	0.9

**Table 2:** Quantitative reconstruction results in the case of two sources for homogeneous phantom experiments.

Noise levels	Number of source	Reconstructed central position using different methods (unit: mm)		
		DA	SP_3_	SP_3_ + Bregman
0%	No. 1	(−5.99, 0.35)	(−5.49, 0.28)	(−5.00, 0.21)
	No. 2	(−0.08, 6.00)	(0.14, 5.50)	(−0.15, 5.00)

10%	No. 1	(−5.99, 0.35)	(−5.49, 0.28)	(−5.00, 0.21)
	No. 2	(−0.08, 6.00)	(0.14, 5.50)	(−0.15, 5.00)

30%	No. 1	(−5.45, 0.78)	(−5.49, 0.28)	(−5.00, 0.21)
	No. 2	(−0.86, 5.43)	(−0.64, 4.96)	(0.07, 4.50)

**Table 3:** Optical property parameters used in the case four sources [29].

Wavelength	*μ*_*a*_ (mm^−1^)	*μ*_*s*_ (mm^−1^)	*μ*_*s*_^'^/*μ*_*a*_	*g*
580 nm	0.463	9.75	2.11	0.9
620 nm	0.107	9.22	8.62	0.9

**Table 4:** Quantitative results between the actual and the reconstructed source centers with different methods in the case of four sources.

Actual source position	Reconstructed source position with SP_3_ method	Reconstructed source position with SP_3_ and Bregman method
(5, −5)	(5.14, −5.47)	(4.96, −4.94)
(−5, −5)	(−5.27, −5.34)	(−4.94, −4.96)
(−5, 5)	(−4.81, 5.76)	(−4.95, 4.95)
(5, 5)	(4.86, 5.72)	(4.95, 4.95)

(5, 0)	(4.98, 0.50)	(4.98, 0.50)
(−5, 0)	(−5.45, 0.78)	(−5.49, 0.28)
(0, 5)	(−0.36, 5.49)	(0.14, 5.50)
(0, −5)	(0.72, −5.96)	(0.58, −5.47)

**Table 5:** Optical properties of biological tissues for different wavelengths [21].

	620 nm			700 nm		
	*μ*_*a*_ (mm^−1^)	*μ*_*s*_ (mm^−1^)	*g*	*μ*_*a*_ (mm^−1^)	*μ*_*s*_ (mm^−1^)	*g*
Muscle	0.086	4.29	0.9	0.0027	11.8	0.9
Skeleton	0.06	24.95	0.9	0.039	23.4	0.9
Heart	0.058	9.63	0.9	0.038	9.05	0.9
Bladder	0.086	4.29	0.9	0.0027	11.8	0.9
Testis	0.086	4.29	0.9	0.043	21.09	0.9
Pancreas	0.345	6.78	0.9	0.23	6.48	0.9
Spleen	0.345	6.78	0.9	0.0077	13.77	0.9
Stomach	0.086	4.29	0.9	0.23	6.48	0.9
Liver	0.345	6.78	0.9	0.043	21.09	0.9
Kidneys	0.05	5.4	0.9	0.23	6.48	0.9
Lungs	0.195	21.73	0.9	0.13	21.24	0.9
